# Effect of occupational exposure to cytostatics and nucleotide excision repair polymorphism on chromosomal aberrations frequency

**DOI:** 10.2478/v10102-009-0002-6

**Published:** 2009-03

**Authors:** L'udovít Mušák, Veronika Poláková, Erika Halašová, Oto Osina, Ludmila Vodičková, Janka Buchancová, Henrieta Hudečková, Pavel Vodička

**Affiliations:** 1 Institute of Medical Biology, Jessenius Faculty of Medicine, Comenius University in Martin, Slovakia; 2 Clinic of Occupational Medicine and Toxicology, Jessenius Faculty of Medicine, Comenius University in Martin, Slovakia; 3 Institute of Experimental Medicine Academy of Sciences in Prague, Czech Republic; 4 National Institute of Public Health in Prague, Czech Republic; 5 Institute of Public Health, Jessenius Faculty of Medicine, Comenius University in Martin, Slovakia

**Keywords:** chromosomal aberrations, cytostatics, occupational exposure, DNA repair genes *XPD*, *XPG*, *XPC*, polymorphisms of genes

## Abstract

Authors evaluated the incidence of total chromosomal aberrations (CA) and their types – chromatid-type (CTA) and chromosome-type (CSA) in peripheral blood lymphocytes from 72 oncologic unit's workers occupationally exposed to cytostatics in relationship to polymorphisms of DNA repair genes *XPD*, *XPG* and *XPC*. The cytogenetic analysis was used for determination of chromosomal aberrations frequency and PCR-RFLP method for polymorphisms of genes. Statistically higher frequency of total CA was detected in exposed group as compared to control (1.90±1.34% vs. 1.26±0.93%; Mann-Whitney U-test, *p*=0.001). There was not detected any difference between CTA and CSA (0.92±1.04% vs. 0.98±1.17%). Similarly, in genes *XPD* exon 23 and *XPC* exon 15 wasn't detected any difference neither in total chromosomal aberrations nor in CTA and CSA types. Statistically significant decrease of total chromosomal aberrations and CTA-type with presence of variant allele C was detected in gene *XPG* exon 15. Authors pointed out the importance of individual susceptibility factors in evaluation of effects of genotoxic agents, in that event, when the concentration does not meet the occupational exposure limit.

## Introduction

Workers in oncologic units are regularly, in the long term occupationally exposed to low doses of cytostatics. There are agents with antineoplastic effect, which are used in chemotherapy in malignant tumour's therapy. These agents have direct toxic or cytotoxic effect, and can act as an important risk factors through their indirect impact (mutagenic, carcinogenic and teratogenic). Antineoplastic agents forestall in irregular division of cells, damage intercellular surroundings and induce the cell's death. Connor (2006) reports that the skin is the first meeting point of antineoplastic agents' contamination. The contamination of the skin was repeatedly discovered even the protective gloves were used (Fransman *et al*., 2005). Fransman *et al*. (2007b) followed also the contamination of bed sheets by antineoplastic drugs (cyclophosphamide, ifosfamide, methotrexate, 5-fluorouracil, etoposide, cytarabine, gemcitabine and chlorambucil). Mentioned cytostatics were found on workers' skin of hands or in any of the air samples. The increased frequency of chromosomal aberrations, sister chromatid exchanges (SCE) and micronuclei (MN) after occupational exposure was described by many authors (Pilger *et al*., 2000; Burgaz *et al*., 2002; Musak *et al*., 2006). Maluf and Erdtmann (2000) learned higher incidence of MN and positive comet assay. Rubes *et al*., (1998) detected noticeable higher number of translocations and unstable CA. Major *et al*. (1999) determined evidently higher frequency of premature divided centromeres. Burgaz *et al*. (2002) discovered 2.5-times higher number of CA within exposed workers in comparison to unexposed persons. Exposure to methotrexate (MTX) caused detectable but slight increase of MN (Deng *et al*., 2005). Rekhadevi *et al*. (2007) and Cavallo *et al*. (2007) detected positive comet assay test, higher frequency of micronuclei in bucal mucosa and peripheral blood and cyclophosphamide (CP) level in urine. Musak *et al*. (2006) published the higher frequency of CA in variant type of alleles in genes *XRCC1* exon 10 and *XRCC3* exon 7. Testa *et al*. (2007) found out not significant relationship between polymorphisms of genes for GST and higher frequency of CAs. Tuimala *et al*. (2002) detected higher number of CTA-type aberrations in peripheral lymphocytes in individuals with variant allele in gene *XRCC1*, codon 280 exposed to bleomycine. The impact of mitomycine C (MMC) a bleomycine (BLM) in terminal segments of chromosomes was evaluated by measure of telomere length. Exposure to MMC cases an important shortening of the following chromosome arms l2q, 3p, 5q, 7p, 10q, 11p, 13q, 17p, 18pq and 21q (Wick and Gebhart, 2005). After exposure to bleomycine (BLM) was detected important influence in genotype *GSTT(–)*, also of polymorphism of gene *XPD* in healthy individuals (Angelini *et al*., 2008). Indirect determination of efficiency of DNA repair mechanisms was stated on the frequency of CAs induced by bleomycine (BLM). Laczmanska *et al*., (2007) published the correlation between CAs frequency and polymorphisms of DNA repair genes for base excision repair (BER) – *XRCC1*, nucleotide excision repair (NER) – *XPA, XPC, XPG, XPD, XPF, ERCC1* and homologous recombination (HR) – *NBS1, RAD51, XRCC2, XRCC3, BRCA1*. Only the gene *XPC* exon 15 and intron 11 was found to be in the relationship with increased frequency of CAs induced by BLM. It point out on importance of NER in CAs reparation. Rombaldi *et al*. (2009) analysed the genotoxicity using comet assay, and MN test as well as the level of oxidative stress. The exposed workers presented increased DNA damage levels by the comet assay as compared to the controls. The comet assay results have also shown significant positive correlation with the length of exposure and with the amount of consumed alcohol. MN frequency was significantly higher in the exposed workers and presented noteworthy correlation with age and working time. In the oxidative stress parameters, only CAT presented a significant increase.

Cytostatics genotoxicity is expressed especially by undirected damage of genetic material. The most of mutagens are electrophilic agents, or can be metabolised by electrophilic intermediators. There are able to create covalent bonds with nucleophilic structures of cells. Cells dispose of mechanism for elimination of genotoxic effect on DNA as well as for reparation of created lesions. The terminal manifestation of mutation is the results of primary genetic damage and the effect of repair processes in cell. Individual efficiency in repair process can be one of primary attribute for inherited predisposition to tumours creation.

## Material and methods

In the present work 148 exposed and control individuals were analysed for frequency of chromosomal aberrations and polymorphisms of DNA repair genes. All of them completed anamnestic questionnaire about length and way of exposure, job categorize, exogenous factors (smoke, drug usage, exposure to radiation, alcohol consumption and dietary) before blood collection and give an agreement to be involved in the study.

Exposed group consists 72 workers from specialized oncologic departments occupationally exposed to cytostatics. They were from three hospitals in north part middle Slovakia region. 28 workers were from Faculty Hospital in Martin, 31 from Central Military Hospital in Ružomberok and 13 from Hospital with polyclinic in Trstená. All of workers were regularly in contact with cytostatics that dilute and apply to patients. By job grade are they nurses and physicians. There were predominantly females in both groups. Smokers form 26.39%. Control group consisted from medical workers and workers from factory Biotika. They were not exposed to any genotoxic agents. Characteristics of exposed and control groups are present in Table 1. We microscopically analysed 100 mitoses per person. We evaluated the frequency of total chromosomal aberrations, and then subdivided them to CTA and CSA types. Methodology of cytogenetic analysis was performed according to AHEM (2007). Polymorphisms of DNA repair genes were performed by PCR-RFLP. Amplificied fragments were pre-digested by restriction endonucleases and analyzed. Genotypes were determined in direct process sequence of amplificied fragments. Gene *XPD* exon 23 (A→C), kodon 751 (Lys751Gln): primers – F (forward): 5′-CCC CTC TCC CTT TCC TCT GTT-3′; R (reverse): 5′-GCT GCC TTC TCC TGC GAT TA-3′; restriction enzyme *Pst*I; size of fragments: 1) normal homozygote (Lys751Lys) – 290+146 bp; 2) heterozygote (Lys751Gln) – 290+227+146+63 bp; 3) variant homozygote (Gln751Gln) – 227+146+63 bp.

**Table 1:** Characteristics of exposed groups and control.

	Exposed group	Control
Number	72	76
Age (years±S.D.)	41.19±8.95	35.99 ±7.73
Exposure (years±S.D.)	11.31±8.98	
Sex (N) M/F	7/65	16/60
Smoking (N) S/NS	19/53	15/61
Job (N) physician/nurse	14/58	20/56

Gene *XPG* exon 15 (G→C), kodon 1104 (Asp1104His): primers – F (forward): F: 5′-TGG ATT TTT GGG GGA GAC CT-3′; R (reverse): 5′-CGG GAG CTT CCT TCA CTG AGT-3′; restriction enzyme *Hsp*92II; size of fragments: 1) normal homozygote (Asp1104Asp) – 159 bp; 2) heterozygote (Asp1104His) – 59+100+159 bp; 3) variant homozygote (His1104His) – 59+100 bp. Gene *XPC* exon 15 (A→C), kodon 939 (Lys939Gln): primers – F (forward): 5′-GAT GCA GGA GGT GGA CTC TCT-3′; R (reverse): 5′-GTA GTG GGG CAG CAG CAA CT-3′; restriction enzyme *Pvu*II; size of fragments: 1) normal homozygote (Lys939Lys) – 281 bp; 2) heterozygote (Lys939Gln) – 150+131+281 bp; 3) variant homozygote (Gln939Gln) – 150+131 bp. In this paper are accepted all principles for protection personnel data, for health care. The design of the study was approved by the Ethical Committee of Jessenius Medical Faculty in Martin.

The peripheral blood sampling was realized within the specialised medical examinations.

Statistical analysis was performed using program Statgraphics, version 7 (Manugistics, Cambridge, MA). We used nonparametric Mann-Whitney U-test for testing differences between groups and analysis of variance (ANOVA) for testing relationships between biomarkers and genotype. The values in tables are presented as average ± S.D.

## Results

**Figure 1:**
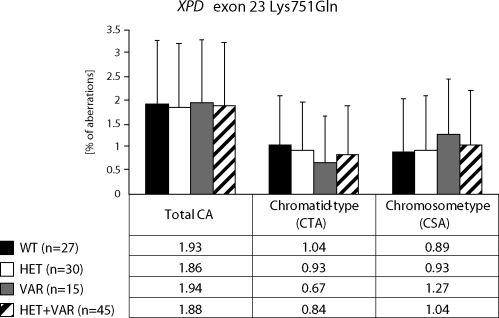
Polymorphisms of XPD gene – total chromosomal aberrations, chromatid-type and chromosome-type of aberrations.

**Figure 2:**
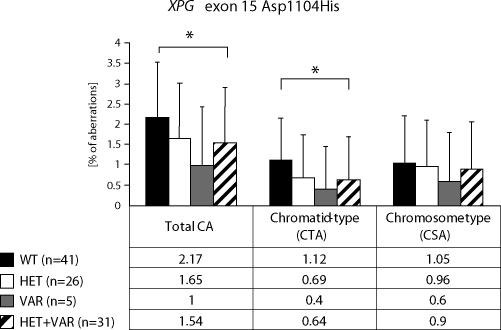
Polymorphisms of XPG gene – total chromosomal aberrations, chromatid-type and chromosome-type of aberrations.

**Figure 3:**
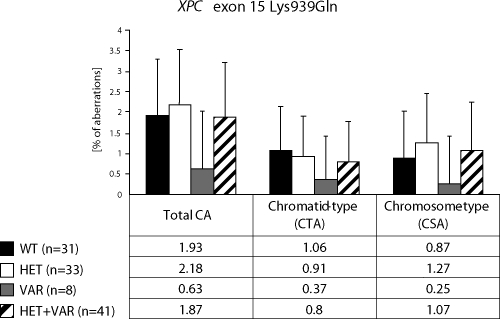
Polymorphisms of XPC gene – total chromosomal aberrations, chromatid-type and chromosome-type of aberrations.

We detected higher frequency of total chromosomal aberrations (CAs) in exposed group in comparison to control (1.90±1.34% vs. 1.26±0.93%, Mann-Whitney U-test, P=0.001). In exposed group we did not detect any difference between chromatid-type (CTA-type) and chromosome-type (CSA-type) – 0.92±1.04% vs. 0.98±1.17% (Table 2). Evaluating the role of *XPD* gene, exon 23 we stated, that the exposition did not influence the total CAs. The frequency of chromatid-type aberrations was faintly lower in the presence the variant allele and contrary, the frequency of chromosome-type aberrations was faintly higher. The differences were not statistically significant (Figure 1). *XPG* gene exon 15 polymorphisms showed the significant decrease of total CAs and CTA-type aberrations frequency if the variant allele was present (*p*<0.05), the frequency of CSA-type aberrations was lower but not significant (Figure 2). In *XPC* gene exon 15 we observed similar trends like in gene *XPD*; i.e. in frequency of total CAs we did not detect any difference between alleles (Figure 3).

**Table 2:** Number of total chromosomal aberrations, chromatid-type (CTA) and chromosome-type (CSA) inexposed group and control.

	Total CA	Chromatid-type (CTA)%±S.D.	Chromosome-type (CSA)%±S.D.
Exposed group	1.90±1.34^***^	0.92±1.04	0.98±1.17
Control	1.26±0.93	0.53±0.62	0.73±0.81

^***^ *p*=0.001 Total CA exposed vs. control

## Discussion

Cytostatics are mutagenic and carcinogenic agents (Vorlíček *et al*., 2000). They cause variously DNA damages. Burgaz *et al*. (2002) followed the effect of 9 cytostatics and Cavallo *et al*. (2005) of 5 cytostatics in nurses and pharmaceutical workers that prepared, diluted or applied cytostatics. The authors examined also workers that were in contact with contaminated bed sheets, possibly excrements of treated patients. They both observed the higher frequency of CAs and MN in buccal mucosa cells in exposed workers. Burgaz *et al*. (2002) detected 2.5-time higher frequency of CAs in exposed persons.

Our findings are in accordance with previously mentioned; we detected higher frequency of total chromosomal aberrations (CAs) in exposed group in comparison to control. However, we did not find any difference between chromatid-type (CTA-type) and chromosome-type (CSA-type) of aberrations in exposed group. Numerous authors determined in their explorations that the increased risk to exposure is related with inconsistent observance of safety regulations. The higher exposures to cytostatics were observed at workplaces, where the personnel were not adequately informed about risk associated with this job and where were not strictly keep the safety directives (Maluf and Erdtmann, 2000; Connor, 2006; Fransman *et al*., 2007a). The human genome is permanently repaired protecting the cells and organism life. It is proved that the DNA repair can influence the CAs frequencies (Skjelbred *et al*., 2006). We can presume certain selection drift of heterozygous in comparison to homozygous. The interest in polymorphisms that are related with modifications in human genome is permanent. There are many discussions pointed on gene-environmental interaction that lead to formation of disease in some individuals. To determine the CAs frequency in human lymphocytes we analysed specific polymorphisms of DNA repair genes. We looked for the relation between genes polymorphism for nucleotid excision repair *XPD*, *XPG* and *XPC and* CAs frequency. In genes *XPD* and *XPC* we did not detect any difference neither in frequency of total CAs nor in their separate types. In gene *XPG* we found out statistically significant decrease of total CAs and CTAs with presence of variant allele. The decrease of CSA-type was not statistically significant. The polymorphism of many genes included in BER or NER, and repair of double strand breaks is tightly connected with increased risk of tumours and DNA damages (Hemminki *et al*., 2001, Hou *et al*., 2002, Kumar *et al*., 2003). Genotoxic impact of gamma radiation reduces the ability of DNA repair process (Sudprasert *et al*., 2006). The reduced ability of DNA repair was detected after exposure to UV light and mitomycine C in malignant cells of prostate. Variant alleles AA of gene *XRCC1* in codon 280 and alleles TT in codon 194 tightly connected with increased ability of the repair DNA chain that was detected in urban population (Tuimala *et al*., 2002). Gene *XRCC3* is needed in repair of breaks by homological recombination and its polymorphism probably decrease the number of sister chromatid exchanges and chromosomal aberrations (Tuimala *et al*., 2004). These authors did not confirm the association between polymorphism of gene *XRCC3* in codon 241 (Thr241Met) and frequency of chromosomal aberrations after treatment of bleomycine. The sensitivity of bleomycine depends on individual ability to DNA repair and is particularly impacted by genetic polymorphism of *XRCC1* gene that affect on DNA repair *in vitro* (Tuimala *et al*., 2002; Tuimala *et al*., 2004). Vodička *et al*. (2004) evaluated the relationship between polymorphisms of DNA repair genes *XRCC1*, *XRCC3*, *XPD, XPG* and *XPC* and frequency of CAs and SCE. They detected the higher frequency of CAs in individuals with allele A in *XPD* gene exon 23, e.i. in individuals with genotype AA a AC. Musak *et al*. (2006) detected higher frequency of CAs in variant type of alleles in genes *XRCC1* exon 10 and *XRCC3* exon 7. Since polymorphisms influence many metabolic processes and detoxification of toxic chemical agents, DNA changes could increase the risk of cancer.

Relatively insufficiently is examined the part of interactions of DNA repair genes. It is caused by the fact, that in DNA repair process are present more than 100 genes, and almost 40 are polymorphic (Mohrenweiser *et al*., 2002). Currently is not sufficient explanation for the relationship between genes for xenobiotics metabolism, DNA repair genes and their function, the manifestation in phenotype. The great attention is spending in genotypes combinations, their inter-genes interactions in evaluation of genotoxic effects of agents in exposed individuals.

From literature is known, that variously genetic polymorphisms can have the influence on the incidence of variety diseases, and particularly tumours and also on genotoxic effects induced by occupational exposure to genotoxic agents (Norppa, 2003; Norppa, 2004). There was optimal to evaluate the individual susceptibility, and to determine “positive” and “negative” genotypes in order to minimalize the risk of exposure in sensitive individuals. It is not possible because we have insufficient informations about genotypes, and interactions of genes.

We evaluated the frequency of total chromosomal aberrations and individual type of them, e.i. CTA-type and CSA-type of workers in oncologic units occupationally exposed to antineoplastic agents in compared to polymorphism of DNA repair genes for nucleotid excision repair *XPD, XPG* and *XPC*. Presented results refer to importance of individual susceptibility's factors in assessment of genotoxic effects, in these cases, when concentration of genotoxic agents usually not exceeds the occupational exposure limit.

## References

[CIT0001] AHEM (2007). Metody biologického monitorování genotoxických účinků faktorů prostředí. Cytogenetická analýza periferních lymfocytů. Aktualizácia platnej štandardnej metodiky.

[CIT0002] Angelini S, Kumar R, Carbone F, Bermejo JL, Maffei F, Cantelli-Forti G, Hemminki K, Hrelia P (2008). Inherited susceptibility to bleomycin-induced micronuclei: correlating polymorphisms in *GSTT1, GSTM1* and DNA repair genes with mutagen sensitivity. Mutat Res.

[CIT0003] Burgaz S, Karahalil B, Canhi Z, Terzioglu F, Ancel G, Anzion RB, Bos RP, Hüttner E (2002). Assessment of genotoxic damage in nurses occupationally exposed to antineoplastics by the analysis of chromosomal aberrations. Hum Exp Toxicol.

[CIT0004] Cavallo D, Ursini CL, Omodeo-Sale E, Iavicoli S (2007). Micronucleus induction and FISH analysis in buccal cells and lymphocytes of nurses administering antineoplastic drugs. Mutat Res.

[CIT0005] Cavallo D, Ursini LC, Perniconi B, Di Francesco A, Giglio M, Rubino FM, Marinaccio A, Iavicoli S (2005). Evaluation of genotoxic effects induced exposure to antineoplastic drugs in lymphocytes and exfoliated buccal cells of oncology nurses and pharmacy employees. Mutat Res.

[CIT0006] Connor TH (2006). Hazardous anticancer drugs in health care: environmental exposure assessment. Ann N Acad Sci.

[CIT0007] Deng H, Hang M, He J, Wu W, Jin L, Zheng W, Lou J, Wang B (2005). Investigating genetic damage in workers occupationally exposed to methotrexate using three genetic end-points. Mutagenesis.

[CIT0008] Fransman W, Huizer D, Tuerk J, Kromhout H (2007a). Inhalation and dermal exposure to eight antineoplastic drugs in an industrial laundry facility. Int Arch Occup Environ Health.

[CIT0009] Fransman W, Roeleveld N, Peelen S, de Kort W, Kromhout H, Heederik D (2007b). Nurses with dermal exposure to antineoplastic drugs: reproductive outcomes. Epidemiology.

[CIT0010] Fransman W, Vermeulen R, Kromhout H (2005). Dermal exposure to cyclophosphamide in hospitals during preparation, nursing and cleaning activities. Int Arch Occup Environ Health.

[CIT0011] Hemminki K (2001). *Biomarkers of exposure and effect for carcinogenicity*. Appendix I. WHO Library Cataloguing-in-Publication Data, Biomarkers in risk assessment: validity and validation (Environmental health criteria) 222.

[CIT0012] Hou SM, Fält S, Angelini S, Yang K, Nyberg F, Lambert B, Hemminki K (2002). The *XPD* variant alleles are associated with increased aromatic DNA adduct level and lung cancer risk. Carcinogenesis.

[CIT0013] Kumar R, Höglund L, Zhao CH, Försti A, Snellman E, Hemminki K (2003). Single nucleotide polymorphisms in the *XPG* gene: determination of role in DNA repair and breast cancer risk. Int J Cancer.

[CIT0014] Laczmanska I, Gil J, Karpinski P, Stembalska A, Trusewicz A, Pesz K, Ramsey D, Schlade-Bartusiak K, Blin N, Sasiadek MM (2007). Polymorphism in nucleotide excision repair gene XPC correlates with bleomycin-induced chromosomal aberrations. Environ Mol Mutagen.

[CIT0015] Major J, Jakab MG, Tompa A (1999). The frequency of induced premature centromere division in human populations occupationally exposed to genotoxic. Mutat Res.

[CIT0016] Maluf SW, Erdtmann B (2000). Follow-up study of the genetic damage in lymphocytes of pharmacists and nurses handling antineoplastic drugs evaluated by cytokinesis-block micronuclei analysis and single cell gel electrophoresis. Mutat Res.

[CIT0017] Mohrenweiser HW, Xi T, Vazquez-Matias J, Jones IM (2002). Identification of 127 amino acid substitution variants in screening 37 DNA repair genes in humans. Cancer Epidemiol Biomarkers Prev.

[CIT0018] Musak L, Vodicka P, Klimentová G, Soucek P, Hánová M, Mikulková R, Buchancová J, Vodicková L, Poláková V, Péc M (2006). Chromosomal damage and polymorphisms of DNA repair genes *XRCC1* and *XRCC3* in workers exposed to cytostatics. Neuro Endocrinol Lett.

[CIT0019] Norppa H (2004). Cytogenetic biomarkers and genetic polymorphisms. Toxicol Lett.

[CIT0020] Norppa H (2003). Genetic susceptibility, biomarker responses, and cancer. Mutat Res.

[CIT0021] Pilger A, Köhler I, Stettner H, Mader RM, Rizovski B, Terkola R, Diem E, Franz-Hainzl E, Konnaris CM, Valic E, Rüdinger HW (2000). Long-term monitoring of sister chromatid exchanges and micronucleus frequencies in pharmacy personnel occupationally exposed to cytostatic drugs. Int Arch Occup Environ Health.

[CIT0022] Rekhadevi PV, Sailaja N, Chandrasekhar M, Mahboob M, Rahman MF, Grover P (2007). Genotoxicity assessment in oncology nurses handling anti-neoplastic drugs. Mutagenesis.

[CIT0023] Rombaldi F, Cassini C, Salvator M, Saffi J, Erdtmann B (2009). Occupational risk assessment of genotoxicity and oxidative stress in workers handling anti-neoplastic drugs during a working week. Mutagenesis.

[CIT0024] Rubes J, Kucharová S, Vozdová M, Musilová P, Zudová Z (1998). Cytogenetic analysis of peripheral lymphocytes in medical personnel by means of FISH. Mutat Res.

[CIT0025] Skjelbred CF, Svendsen M, Haugan V, Eek AK, Clausen KO, Svendsen MV, Hansteen IL (2006). Influence of DNA repair gene polymorphisms of *hOGG1*, *XRCC1*, *XRCC3*, *ERCC2* and the folate metabolism gene MTHFR on chromosomal aberration frequencies. Mutat Res.

[CIT0026] Sudprasert W, Navasumrit P, Ruchirawat M (2006). Effects of low-dose gamma radiation on DNA damage, chromosomal aberration and expression of repair genes in human blood cells. Int J Hyg Environ Health.

[CIT0027] Testa A, Giachelia M, Palma S, Appolloni M, Padua L, Tranfo G, Spagnoli M, Tirindelli D, Cozzi R (2007). Occupational exposure to antineoplastic agents induces a high level of chromosome damage. Lack of an effect of GST polymorphisms. Toxicol Appl Pharmacol.

[CIT0028] Tuimala J, Szekely G, Gundy S, Hirvonen A, Norppa H (2002). Genetic polymorphisms of DNA repair and xenobiotic-metabolizing enzymes: role in mutagen sensitivity. Carcinogenesis.

[CIT0029] Tuimala J, Szekely G, Wikman H, Järventaus H, Hirvonen A, Gundy S, Norppa H (2004). Genetic polymorphisms of DNA repair and xenobiotic-metabolizing enzymes: effects on levels of sister chromatid exchanges and chromosomal aberrations. Mutat Res.

[CIT0030] Vodicka P, Kumar R, Stetina R, Sanyal S, Soucek P, Haufroid V, Dusinska M, Kuricova M, Zamecnikova M, Musak L, Buchancova J, Norppa H, Hirvonen A, Vodickova L, Naccarati A, Matousu Z, Hemminki K (2004). Genetic polymorphisms in DNA repair genes and possible links with DNA repair rates, chromosomal aberrations and single-strand breaks in DNA. Carcinogenesis.

[CIT0031] Vorlíček J, Vyzula R, Adam Z (2000). Praktická onkologie, vybrané kapitoly.

[CIT0032] Wick U, Gebhart E (2005). Studies on the action of mitomycin C and bleomycin on telomere lengths of human lymphocyte chromosomes. Int J Mol Med.

